# Emotional and social repercussions of stroke on patient-family caregiver dyads: Analysis of diverging attitudes and profiles of the differing dyads

**DOI:** 10.1371/journal.pone.0215425

**Published:** 2019-04-23

**Authors:** Barbara Bucki, Elisabeth Spitz, Michèle Baumann

**Affiliations:** 1 Institute for Research on Socio-Economy and health Inequalities (IRSEI), Unit INSIDE, University of Luxembourg, Luxembourg city, Grand-Duchy of Luxembourg; 2 Department of Psychology, EA4360 APEMAC, University of Lorraine, Paris Descartes University, Metz, France; Northern Arizona University, UNITED STATES

## Abstract

For patients, the social and emotional repercussions of stroke include shame, personality changes, and upheavals experienced by the couple (i.e. patient and main family caregiver). These impacts on the couple ‘patient/family caregiver’ are scarcely documented. Focusing on the perceptions of the patients and the family caregivers living at home, two years after a stroke occurrence, the aims of the study were to analyse the concordance of attitudes towards the emotional and social repercussions of stroke and to determine the profiles of the differing dyads. Two researchers conducted separate face-to-face structured interviews with stroke survivors and their family caregivers. Eleven items, identified through a content analysis of interviews and after a qualitative process of generating questionnaire items, assessed the commonly experienced impact of stroke on the family, the social repercussions of stroke, and its emotional effects on the stroke survivors. The kappa concordance coefficient was used to determine the response convergence between patients and family caregivers. Four items, selected by a panel of experts, were included in logistic regressions (i.e., demographic characteristics and patients’ impaired functions) to identify the differing dyadic profiles. Family caregivers’ and patients’ attitudes towards the social repercussions of stroke were similar. Patients with motor deficiencies tended to underestimate the upheaval brought to their couple by stroke, whereas caregivers of language-impaired patients tended to underestimate their feelings of shame and demeaning. Communication disturbances, but also residual physical disabilities in stroke survivors, may affect the understanding of each other’s attitudes within dyads. In order to avoid dysfunctional relationships between family caregivers and patients, healthcare professionals need to pay special attention to this issue, especially in cases of aphasia and motor deficiencies.

## Introduction

Stroke is one of the main causes of acquired disability in most developed countries and considerably affects the life course of the victims [1]. Depending on the affected brain area, stroke can leave long-term impairments of motor, sensory, and/or cognitive functions, but is also associated with social changes [2]. At the occupational level, an average of two out of five stroke victims working at the time of occurrence were able to return to work; at the personal level, stroke can affect the relationships with children and the partners—including the sexual sphere—as well as deteriorate family dynamics and decrease participation in social activities [3, 4].

Since a majority of the survivors return home, their family caregivers, mainly their partners, also experience these social repercussions of stroke. Expected to provide complex care at home in addition to having new responsibilities (increased home tasks and duties, management of relationships with the professionals, etc.), these dependency workers [5] pose a risk for the healthcare system while their exhaustion increases and their cultural, social, and leisure activities decrease [6, 7]. This workload, in addition to their continual adaptation to the limitations of the patient, may isolate them socially. Thus, when they themselves are affected by the repercussions of the stroke as the caregiver, their capacity to remain healthy becomes a challenge for public health and health policies [8, 9].

### Patient-caregiver dyad as a unit in and of itself

A strong association exists between the experiences of the patients and their family caregivers thus allowing the patient-caregiver dyads to be considered as entities [10] based on both feelings and caring [11]. After a stroke event, the quality of the relationship plays a key role on the respective well-being of the patient and the family caregiver.

### The usefulness of identifying closeness of attitudes

Highlighting the concordance between patients and caregivers is challenging: studies on mutual trust [12] and harmony in social relations [13] have shown that when stroke patients and their family caregivers share similar opinions, the psychological health of patients is higher. In couples, the positively perceived quality of communication is generally associated with higher satisfaction of partners with their relationship [14] and helps to enhance the dyadic adjustment during a chronic illness [15]. Yet, while the patient-caregiver relationship should be considered as a balance between giving and taking [16], social support appears to be rather unidirectional, favouring the patient [17]. Thus, there is a need to investigate the link between patients’ and family caregivers’ attitudes towards a situation commonly shared, such as the social repercussions of stroke.

In this study, emphasis was made on the concordance of three impacts of stroke on patients and their family caregivers: changes in the patient’s personality and emotions due to stroke, effects of stroke on the family members, and the extended social repercussions of stroke.

### Change in personality of stroke survivors and its impact on family caregivers

Personality change of the stroke patient is one of family caregivers’ recurring complaints [18], resulting either directly from stroke sequelae [18] or reflecting the stroke survivor’s way of adapting to this potentially traumatic event [11, 19].

Detecting personality change is particularly useful since affective disorders have been shown to play a more important role than motor impairments or pain on their family caregivers’ quality of life and satisfaction with life [6]. Moreover, family caregivers reporting personality changes in their stroke relatives have more frequently reported experiencing emotional distress [18].

### Emotions felt and shared by stroke victims

Being a stroke survivor triggers a multitude of emotions. Bitterness, shame, and grief are commonly reported after the onset of a stroke [20]. Some patients feel relief that its severity was not worse; recovery gains instil pride [21]. Although sharing one’s emotions allows understanding and learning from difficult situations and helps strengthen social bonds [22], shame is one of the least shared emotions [23]. In addition, to protect their family caregivers, some patients tend to minimise the impact severity of stroke [24]. As a result, the ability of the family caregivers to detect their relatives’ ill-being and, furthermore, to support them is made more difficult.

To evaluate the potential interventions that would decrease the impact of stroke on dyadic communication, there is a need to determine the extent to which the attitudes of dyad members differ with regard to the emotional effects of stroke on the survivors.

### Attitude concordance within dyads

Concordance is the agreement between the attitudes of two persons. Most of the literature on concordance has assessed the reliability of resorting to caregivers as proxy respondents for patients with limited ability due to their chronic illness [25–28]; in these studies, caregivers tend to overestimate patient symptoms and underestimate patient quality of life.

No previous literature has explored the attitudes of both patients and their caregivers regarding the social repercussions of stroke, experienced jointly during the chronic phase, nor has it identified the profiles of the differing dyads. Three important aspects reflect the quality of the dyadic interactions between stroke patients and their caregivers: (1) concordant attitudes resulting from prior communication, (2) concordant attitudes regarding commonly experienced psychosocial impacts of stroke, and (3) the psychological repercussions experienced by the stroke patients.

### Objectives

Focusing on the perceptions of the stroke survivors and their family caregivers living at home two years after the stroke occurrence, the aims of the study were to analyse the concordance of attitudes on the emotional and social repercussions of stroke and to determine the differing dyadic profiles.

## Materials and methods

**Fig 1:**
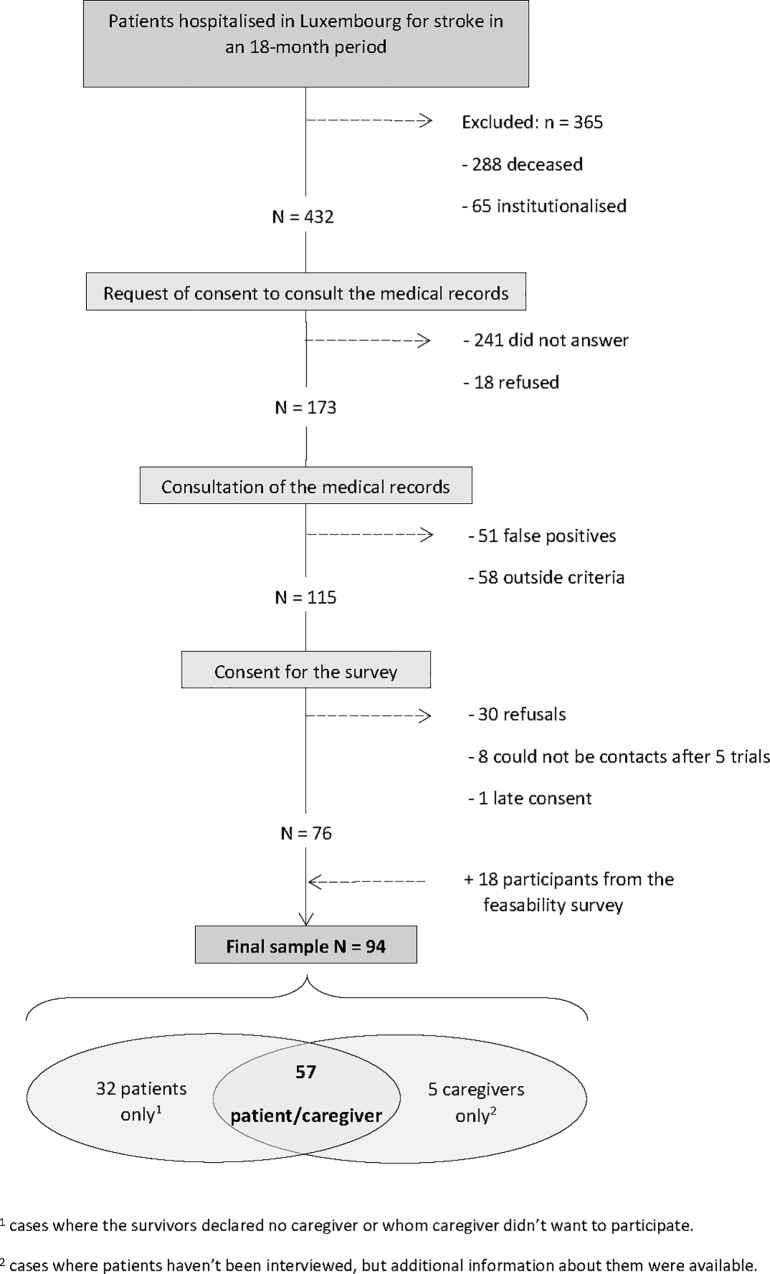
Description of the sampling process.

This study is part of a larger national survey on living at home two years after stroke in Luxembourg, conducted among all adult (≥18 years) survivors of stroke and their main family caregivers. Over a period of 18 months, the records of all the patients hospitalised two years earlier in Luxembourg (N = 797) were analysed (e.g., Fig 1).

Their status—living or deceased—was obtained from the Civil Status Registry. Survivors were drawn from the list compiled by the ‘*Inspection Générale de la Sécurité Sociale*’ of Luxembourg. Among the 432 eligible persons (living, not institutionnalised, residing in Luxembourg, contact information available), 173 gave their informed consent to have their medical records consulted by a practitioner investigator of the Research Centre for Health in Luxembourg. This step allowed to confirm the stroke diagnosis in 115 cases (the others were false positives or did not complete the clinical criteria of stroke). When returning an informed consent form to participate to a face-to-face interview, these were asked to identify their main caregivers as ‘*the person[s] who mostly takes care of [them] since the stroke’*. The final sample was composed of 94 participants, including 57 stroke survivor / caregiver dyads. Appointments was organised at their homes. Two researchers separately conducted face-to-face structured interviews supported by two distinct questionnaires: one for the survivors and one for the caregivers.

### Ethical aspects

The protocol was approved by the National Committee of Research Ethics (CNER) and reported to the Committee for Data Protection of Luxembourg.

### Data collected from both stroke survivors and family caregivers

The attitudes towards the most frequently experienced emotional and social repercussions of stroke were assessed by 11 items [6] identified through a content analysis of 10 interviews and after a qualitative process of generating questionnaire items [29]. The items measured three effects of stroke on the patient (*character change*, *feeling demeaned*, *and feeling ashamed to see friends*), four impacts on the family (*whether stroke led to an upheaval in the family and in the couple*, *children distancing*, *and strengthening of family bonds*), and four extended social repercussions (*whether they received expressions of sympathy*, *whether friendship bonds were strengthened*, *whether they lost friends*, *and whether their social life was maintained as before*). The attitudes were measured on a 4-point Likert scale ranging from ‘*totally disagree*’ to ‘*completely agree*’.

The following socio-demographic characteristics were assessed: age, sex, educational level (below *12th grade; 12*^*th*^ *grade and above*), occupational activity at the time of stroke onset (*never employed; manual worker; employee/executive manager/technician; manager/liberal profession*), changes in financial situation due to stroke (yes; no).

### Data collected from stroke survivors

Neurological impairments of the motor, sensory, and visual functions, incontinence, and memory and language impairments were assessed, as identified by the American Heart Association Stroke Outcome Classification (AHA.SOC) [30]. The number of affected domains was expressed in four categories: *‘no impaired domain’*, *‘one impaired domain’*, *‘two impaired domains’*, *and ‘more than two impaired domains’*.

### Statistical analysis

The items assessing the emotional and social repercussions of stroke were dichotomised into ‘agree’ and ‘disagree’. The response distribution was analysed using percentages, means, and standard deviations when appropriate. The concordance between patients’ and caregivers’ attitudes was measured using the kappa concordance coefficient. A significant kappa indicates that the response distribution is not due to chance. A non-significant kappa indicates that there are substantial discordance among the responses, but does not provide information regarding the type of discordance—do patients tend to agree and caregivers disagree or vice versa versus both patients and caregivers agreeing or disagreeing at the same frequency? In order to complete the discordance analysis, a panel of experts composed of psychologists, neurologists, and members of the research unit jointly selected items to be analysed more deeply to illustrate the situation [29]. After presenting the response distribution, a group of discordant dyads was isolated by creating a binary variable (i.e. ‘caregiver agrees and patient disagrees’ vs. ‘others’) and entered as the dependent variable of a logistic regression. This method allowed us to identify the weight of each adjusted independent variable relative to the others. The independent variables were sex and age of the patients and the caregivers, the patients’ impaired motor, visual, and sensory functions, incontinence, and residual language and memory disabilities.

## Results

Among the 57 patient-caregiver dyads, one patient did not respond to any of the items; thus, the sample size decreased to 56 dyads. Of these, 50 dyads were composed of couples (89.3%), and 32 (64%) had wife caregivers and 18 (36%) husband caregivers. In the six remaining dyads, five caregivers were women (83.3%), mainly daughters caring for a parent, and one was another family member—not specified. A majority of households had completed at least 12 years of education.

### Profiles of the respondents and the dyads

Two-thirds of the affected individuals were men, with an average age of 63.3 years. Two years after stroke occurrence, 50% had residual sensory impairments, primarily pain. Nearly one-third had language and memory disorders, and 39.3% had sustained motor deficiencies. Nearly 43% of stroke survivors reported three or more impaired domains.

Among the caregivers, two-thirds were women, with an average age of 59 years. About 90% of these were the patients’ spouses. Results are detailed in Table 1.

**Table 1:** Socio-demographic and health characteristics of the 56 dyad members.

				Patients			Caregivers		
				N	%		n	%	
Sex		Women		21	37.5		36	64.3	
		Men		35	62.5		20	35.7	
Relationship to patient		Spouse					50	89.3	
		Child					5	8.9	
		Other					1	1.8	
Educational level		Below 12^th^ grade		25	44.6		24	42.9	
		12^th^ grade and above		31	55.4		32	57.1	
Residual impairment domains		Motor		22	39.3				
		Vision		12	21.4				
		Sensory		28	50.0				
		Language		18	32.1				
		Memory		21	37.5				
		Character		9	16.1				
		Incontinence		6	10.7				
Number of deficiencies		0		13	23.2				
		1		10	17.9				
		2		9	16.1				
		3 or more		24	42.9				
Age (mean, sd)				63.3 (15.3)			59.1 (13.8)		

### Attitudes regarding emotional and social repercussions of stroke

#### Response distribution

As shown in Table 2, a majority of patients and caregivers reported that stroke had helped strengthen their bonds with their friends: They not only received expressions of sympathy, they were also able to maintain their social life as before the stroke.

**Table 2:** Patients' and caregivers' attitudes towards stroke repercussions and dyad concordance.

	Patients (n = 56)		Caregivers (n = 56)				
	Agree	Disagree	Agree	Disagree	Concordance		
	%	%	%	%	%	K	P
**Social repercussions**							
1. Upheaval in the family	55.4	44.5	64.3	35.7	76.8	0.52	0.000***
2. Upheaval in the couple	22.6	77.4	38.9	61.1	62.9	0.39	0.002**
3. Distance with children	08.2	91.8	09.8	90.2	66.1	0.14	0.319
4. Expressions of sympathy	83.9	16.1	75.9	24.1	71.0	0.43	0.001***
5. Family bonds strengthened	66.1	33.9	69.2	30.8	54.8	0.21	0.120
6. Friend bonds strengthened	87.3	12.7	90.9	09.1	71.0	0.07	0.623
7. Loss of friends	09.3	90.7	08.9	91.1	79.0	0.39	0.003**
8. Social life maintained	73.2	26.8	80.4	19.6	67.6	0.30	0.020*
**Emotional repercussions on the patient**							
9. Patient feels demeaned	44.6	55.4	25.5	74.5	51.6	0.20	0.121
10. Patient’s personality changed	42.9	57.1	53.6	46.4	64.5	0.43	0.001***
11. Patient ashamed of seeing friends	12.5	87.5	03.6	96.4	79.0	0.18	0.102

Significance levels:

*p<0.05

**p<0.01

***p≤0.001.

In contrast, patients as well as caregivers mostly disagreed with the statements that the patient was ashamed of seeing friends, the stroke had put a distance between them and their children, and they had lost friends.

However, more than half of the caregivers stated that the personality of the affected person had changed vs. 43% of the patients. Nearly 45% of the survivors felt demeaned vs. only 25.5% of their caregivers.

More than 55% of the survivors (vs. 64.3% of the caregivers) felt that the stroke had brought an upheaval in their family while 22.6% (vs. 38.9%) reported that stroke had brought an upheaval in their couple relationship.

#### Concordance

Percentage of concordance between patients and their caregivers’ statements (Table 2) ranged from 51.6% (*patient’s feeling demeaned*) to 79% (*shame of seeing friends* and *loss of friends*).

The most concordant items concerned the attitude towards the upheaval the stroke had brought to the family (κ = .52), the patient’s personality change and expressions of sympathy (κ = .43), and upheaval the stroke had brought to the couple and loss of friends (κ = .39).

In contrast, attitudes of caregivers and patients were less concordant with regard to whether the stroke had strengthened bonds with friends (κ = .07), led to children distancing (κ = .14), and whether the patients felt demeaned (κ = .20).

### Illustration and discordant dyad profiles

Patients’ and caregivers’ responses to the items questioning the patients’ feeling demeaned and ashamed, the patients’ personality change, and upheaval in the couple are shown in Table 3.

**Table 3:** Response distribution for the four illustrative items.

***The patient feels demeaned***				
		Patients		
		Agree	Disagree	Total
Caregivers	Agree	08	05	13
	Disagree	14	24	38
	Total	22	29	51
***The patient is ashamed of seeing friends***				
		Patients		
		Agree	Disagree	Total
Caregivers	Agree	1	01	02
	Disagree	6	48	54
	Total	7	49	56
***The patient’s personality changed***				
		Patients		
		Agree	Disagree	Total
Caregivers	Agree	19	11	30
	Disagree	05	21	26
	Total	24	32	56
***Stroke brought serious upheaval in our couple***				
		Patients		
		Agree	Disagree	Total
Caregivers	Agree	09	11	20
	Disagree	03	30	33
	Total	12	41	53

The results of logistic regressions of these four items on discordant dyads are presented in Table 4.

**Table 4:** Logistic regressions of the discordant groups for the four items.

		*The stroke-affected person* *feels demeaned*			*The patient is ashamed* *to see friends*			*The patient’s personality* *is changed*			*Stroke brought serious upheaval in our couple relationship*		
		B	S.E.	*p*	B	S.E.	*p*	B	S.E.	*p*	B	S. E.	p
Intercept		-2.04	2.91	0.482	-8.92	4.47	0.046	-1.75	4.32	0.686	-1.97	2.11	0.350
Age of the caregiver		-0.06	0.05	0.235	-0.01	0.07	0.930	0.04	0.06	0.432	-0.08	0.06	0.226
Age of the patient		0.03	0.04	0.455	0.03	0.06	0.603	-0.04	0.05	0.442	0.06	0.06	0.272
Sex of the caregiver	Women	1.69	2.12	0.424	2.82	2.80	0.313	0.95	3.91	0.809	-	-	
	Men	-			-			-			-	-	
Sex of the patient	Women	1.17	1.98	0.554	2.31	2.31	0.316	-1.62	3.87	0.675	-	-	
	Men	-			-			-			-	-	
Motor impairment	Yes	0.60	0.90	0.507	0.35	1.37	0.798	0.38	0.95	0.691	2.14	1.00	0.027*
	No	-			-			-			0	-	
Vision impairment	Yes	-0.52	1.05	0.622	0.48	1.18	0.686	-0.38	1.08	0.725	-1.83	1.24	0.142
	No	-			-			-			0	-	
Sensory impairment	Yes	0.28	0.97	0.773	0.63	1.70	0.710	0.71	0.91	0.434	0.10	1.00	0.922
	No	-			-			-			0	-	
Language impairment	Yes	2.11	1.00	0.036*	2.56	1.37	0.063^**#**^	-0.25	1.01	0.808	-1.04	1.09	0.342
	No	-			-			-			0	-	
Memory impairment	Yes	-0.86	1.00	0.393	0.17	1.21	0.887	-1.01	1.03	0.331	1.38	0.88	0.115
	No	-			-			-			0	-	
Incontinence	Yes	2.73	1.47	0.064^#^	-1.37	2.05	0.505	-0.79	1.41	0.574	-	-	
	No	-			-			-			-	-	

Significance levels:

^#^p<0.1

*p<0.05.

#### Demeaning

Among the 22 dyads in which the patients stated that he/she felt demeaned, 14 caregivers disagreed with this assessment. Dyads with a higher probability of reporting this type of discordance were those consisting of stroke survivors suffering from language impairments and incontinence.

#### Shame

Among the seven dyads with patients reporting that they felt ashamed to see friends, only one caregiver corroborated this feeling. Discordance was rather observed in dyads whose patient presented language impairments.

#### Personality change

Even though 30 caregivers reported that the personality of the stroke survivor had changed, 11 of the patients they cared for felt their personality was the same as before the stroke. In our analyses, this discordance type was not associated with any socio-demographic feature or type of impairment of the patient.

#### Couple upheaval

In our sample, 20 caregivers declared that stroke had brought an upheaval in their couple relationship, while 11 of their patients did not agree. This discordance type only occurred in dyads where the caregiver was female and the patient male, and particularly when the patient suffered from motor impairments. No such discordance appeared when the patient was incontinent.

## Discussion

Our study analysed the concordance of attitudes between patients and their caregivers towards the emotional and social repercussions of stroke and determined the differing dyadic profiles. The attitudes of both family caregivers and stroke survivors towards the social repercussions of stroke were similar. Stroke patients with motor deficiencies tended to underestimate the upheaval brought to their couple relationship due to the event, whereas caregivers of language-impaired relatives tended to underestimate their feelings of shame and feeling demeaned. Caregivers generally tended to overestimate the upheaval on the couple and the patients’ personality change. Thus, communication disturbances, but also residual physical disabilities, may affect the understanding of each other’s attitudes within dyads.

Couple upheaval was more of a concern for family caregivers than for patients. Similarly, family caregivers also perceived a change in their stroke-affected relatives’ personality. Our study shows that family caregivers, more than the patients, experience the relationship with the other as discontinuous compared to the pre-stroke period, suggesting that becoming family caregivers can be experienced as a biographical disruption [31] which may impair their sense of coherence and thus alter their health behaviours [32]. Research analysing the distortions between individual and dyadic strategies revealed that ill persons received more social support from their caregiver than the opposite [33]. Our findings may reflect that the caregivers are making noticeable efforts to maintain a normal lifestyle for the stroke survivors. Another possible interpretation of our results is that the patients’ relationship with their partners is not a priority for them because they already have to cope with functional impairments (particularly in the motor dimension), a decrease in their quality of life, socio-economic problems [34], and the fear of suffering a new stroke [35].

Our findings also show that it can be difficult for some family caregivers to detect negative affect (such as shame and feeling demeaned) in stroke patients, especially when the stroke survivors suffer from language impairments. Shame is known to be one of the least shared emotions [36]. In the context of chronic disease, shame can be elicited by moralising health professionals [36] or the weight of social norms. In the latter condition, shame is a response to a social vulnerability to stigma [37, 38], as is reported by patients with Parkinson’s disease or lung cancer. In order to maintain their self-esteem and not add an additional burden of social support to be provided by their caregivers, some affected patients may hide their negative affect in front of others [24]. The frequency of discordant attitudes indicates that shame is an intimate feeling, but also suggests that some stroke survivors are confronted with a stigmatisation of their handicaps/disabilities [6]. Experiencing shame has been shown to have positive effects on the survivors’ motivation towards health behaviours [39]. However, further studies are necessary to identify to what extent keeping this emotion to themselves is actually beneficial or not for their wellbeing, and whether it indicates the quality of the relationship with their partners. On the other hand, one should be attentive to the possibility that some caregivers have set up a negative dyadic coping strategy consisting of not paying attention to the survivors’ affects, as observed in depressed individuals [37]. Our findings should also not obscure the fact that a majority of the patients did not report feeling ashamed in front of friends.

Of the four analysed discordant socio-demographic dyadic profiles, one was strongly related to the gender of caregivers; indeed, gender studies report that discordance regarding the level of quality of life of survivors is higher in couples where the caregiver is a woman [26], and that female spouses tend to be overprotective towards their ill counterpart [38]. The discordance of attitudes towards the upheaval in the couple only occurs in female caregiver/male patient dyads. Based on a feminist approach, this tendency of withholding their own feelings is typically observed in women, as a study describing shame in women with chronic pain has shown [40]. Accordingly, the patients may not even notice the magnitude of the changes. This finding underscores the notion that the caregiving role still has gendered specificities [41].

The social repercussions of stroke affected the survivors and the caregivers similarly. Our results show that while family caregivers do not carry the effects of stroke within them, they are affected by its social impacts. In addition to impairing their physical [42] and psychological [43–45] health, caring can also deteriorate its social dimension. Attending to social networks may require an additional effort on their part, especially when they fear leaving the patients alone [6].

None of the attitudes regarding the social repercussions experienced in common by the survivors and caregivers received total concordance. The recent interest for analysing the patient/caregiver as an entity [11] must not obscure the fact that patients and caregivers perceive their reality individually through their own filters [46]. Can such discordances be beneficial to the dyads’ balance? One can assume that caregivers who identify the accurate emotions of the survivors will probably be better able to respond adequately, which will in turn improve the quality of their relationship. For example, caregivers who do not perceive that their relatives feel ashamed in front of friends can interpret their desire to stay at home as laziness or unwillingness. This competence has been, for example, conceptualised through family caregiving skills, and includes accurate monitoring and interpretation [47].

The dissimilarities in attitudes observed in our study indubitably draw attention on the need to provide information to both survivors and caregivers. Actions addressed to dyads can improve the content of the relationship and the caregivers’ health [48]. Moreover, a high reciprocity and exchange in regard to feelings between patients and their caregivers has been shown to decrease the caregiving burden [49]. Eliciting communication among the dyads could enhance the experience of living after a stroke, for both the patients and their caregivers.

### Limitations

Inherent limitations to our study are linked to the small size of our sample and the high rate of mortality two years post-stroke as well as to the size of the Luxembourg, which is one of the smallest countries in Europe. In particular, it results in low power for the tests performed. We did not made any adjustment to take into account for the multiple tests.

From a methodological standpoint, some limits should be acknowledged, such as the drawbacks of the kappa concordance coefficient. For example, concordance rates of only 63% for the ‘upheaval in the couple’ and ‘patient’s personality change’ were significant. This phenomenon has been described in the literature as a paradox [50, 51]. The microanalyses performed here nevertheless overcame these drawbacks and allowed a more in-depth understanding of our results.

In addition, the Luxembourg national survey did not gather information on comorbidities, especially of mental health—such as comorbid depression, anxiety, and psychological distress—which could explain, at least to some extent, the differences that were obtained here [52]. Therefore, for further research, the consequences of the emotional states of stroke patients and their caregivers should be controlled with respect to mental health variables to enable a more careful interpretation of the results.

### Practice implications

Patient education in the chronic phase of stroke could be improved by addressing dyads as a whole, rather than only one of the members. Initiatives have already been taken in the field of psychological distress, which consist of regular meetings between survivors, caregivers, and health professionals. These ‘trialogues’ help people interact, understand each other, and find innovative solutions to their daily challenges [53].

Our recommendations focus on the need for professionals to accompany survivors and their caregivers in their lives after stroke, in the sense of more reciprocity and mutual comprehension. For this purpose, proposing services aimed at promoting communication of emotions within the dyads would help to harmonise the giving-receiving balance, including facilitating emotion sharing as well as developing the caregivers’ caregiving skills and, empowerment, and to reduce the stigmatizsation [54]. Healthcare professionals should also be attentive to those dyads where the survivors suffer from language impairments, especially in cases of aphasia, since they are more vulnerable to misunderstandings while sharing their emotions.

A satisfactory relationship with an ill partner is associated with less caregiving burden. It is also related to better communication within the couple [55]. Thus, actions aimed at promoting communication between partners are expected to improve the wellbeing of caregivers. Although each partner should have the choice to maintain some level of intimacy, it is the duty of the healthcare and social practitioners to create the best environment possible to promote fruitful exchange within the dyad.

## Conclusions

In the chronic phase following stroke, family caregivers’ and survivors’ attitudes towards social repercussions of stroke are similar. The upheaval felt on the level of the couple was even higher for caregivers than for the survivors. Together, they have to cope with the social isolation potentially incurred by the stroke. Negative affects experienced by the survivors, such as shame and feeling demeaned, are not necessarily perceived by their caregivers, which may be associated with inadequate responses to the survivors’ needs. The dissimilarities in attitudes raise the question as to the quality of the relationship between two relatives where one takes care of the other. Further research may help in finding means to enhance the communication between the members of the dyads, which would help reinforce their respective health capability [56].

## Supporting information

S1 File Author summary. (DOCX)
