# Supplementary material for: Emotional and social repercussions of stroke on patient-family caregiver dyads: Analysis of diverging attitudes and profiles of the differing dyads
Source: PLoS One. 2019 Apr 23;14(4):e0215425. doi: 10.1371/journal.pone.0215425 (PMC6478282; doi:10.1371/journal.pone.0215425)
Supplement: S1 File — (DOCX) [file pone.0215425.s001.docx]

**What is known about this topic:**

- Stroke affects social relationships and produces emotions such as feeling demeaned and shame.
- Family caregivers are affected by the social and emotional repercussions of stroke.
- Communication in the couple enhances quality of partners’ relationships.

**What this paper adds:**

- Emotions felt by stroke patients, such as shame or demeaning, can be underestimated by their main caregivers.
- The upheaval that stroke brings to couples is often underestimated by patients.
- Interventions aiming at helping dyads to communicate better may decrease the impact of the social and emotional repercussions of stroke.
